# Quilt Plots: A Simple Tool for the Visualisation of Large Epidemiological Data

**DOI:** 10.1371/journal.pone.0085047

**Published:** 2014-01-13

**Authors:** Handan Wand, Jenny Iversen, Matthew Law, Lisa Maher

**Affiliations:** The Kirby Institute, University of New South Wales, Sydney, Australia; Imperial College London, United Kingdom

## Abstract

**Background:**

Graphical representation of data is one of the most easily comprehended forms of explanation. The current study describes a simple visualization tool which may allow greater understanding of medical and epidemiological data.

**Method:**

We propose a simple tool for visualization of data, known as a “*quilt plot*”, that provides an alternative to presenting large volumes of data as frequency tables. Data from the Australian Needle and Syringe Program survey are used to illustrate “*quilt plots*”.

**Conclusion:**

Visualization of large volumes of data using “*quilt plots*” enhances interpretation of medical and epidemiological data. Such intuitive presentations are particularly useful for the rapid assessment of problems in the data which cannot be readily identified by manual review. We recommend that, where possible, “*quilt plots*” be used along with traditional quantitative assessments of the data as an explanatory data analysis tool.

## Introduction

Clinical and epidemiological studies frequently collect data on periodically measured characteristics to identify and better understand patterns and trends in risk factors over time in order to target higher risk populations and/or investigate the impact of public health interventions [1], [2]. This large volume of information is typically summarized using numerous quantitative presentations such as frequency tables and other forms of descriptive statistics which require simultaneous comparisons of numbers such as frequencies and percentages in many cells. Traditional approaches to evaluating these types of data usually rely on point-by-point comparisons of observed cell frequencies and percentages. Therefore more practical methods are required to present the results in order to investigate important features of the collective data such as trends, outliers, slow or sudden changes.

When used appropriately, graphical displays provide an easily comprehended form of explanation. A wide variety of graphical tools and software exist to visualize the distribution of data for two categorical variables [3], [4]. In the statistical literature, “*quilt plots*” (also known as “image plots”), have been underutilised for the display of categorical data [5], [6].

In this study, we propose a very simple data analysis tool to convert frequency tables for three categorical variables into a *semi-quantitative* presentation which plots the first variable against the second variable and shades the plot with respect to the third variable. A favourable feature of the “*quilt plot”* is that it does not require colour graphics for improved interpretation. Black and white graphics are sufficient. This allows the same objectives to be achieved through a single, easily digested plot. We provide an example of quilt plots and their efficacy.

A wide variety of graphical tools and software exist to visualize the distribution of data including “*fields*”, primarily used to analyze spatial data and “*heat maps*”, commonly used to analyze data from microarray experiments [3]–[8]. However, these tools have complex functionality and require the specification of many parameters.

In the statistics literature *“quilt plots”* (also known as image plots) have been underutilized for the display of categorical data [9], [10]. “*Quilt plots*” and the relevant computer code presented in this paper are a revised and simplified formulation of the relatively complex function “*heat maps*”. This may be particularly useful for epidemiologists and public health researchers who are not familiar with publicly available software such as the R package and its functions.

## Methods

### Ethics Statement

Ethical approval, including approval to obtain verbal consent from respondents, was obtained from the University of New South Wales, along with other relevant jurisdictional and site specific Human Research Ethics Committees. A one page participant information sheet was used and respondents provided informed verbal consent for their voluntary, anonymous and unreimbursed participation. The study population was comprised of people who inject drugs (PWID), a group who are engaged in an illegal activity.

### Data

We used 1995–2010 data from the Australian Needle and Syringe Program Survey (ANSPS). The ANSPS methodology is described in detail elsewhere [11], [12]. Briefly, the ANSPS is an annually repeated cross-sectional sero survey of human immunodeficiency virus (HIV) and hepatitis C virus (HCV) antibody prevalence and associated risk behaviour conducted among PWID attending needle and syringe programs (NSP). Consenting attendees at ∼50 NSP services across Australia completed a brief self-administered questionnaire on demographic characteristics and injecting and sexual risk behaviour. Respondents also provided a capillary dried blood sample which was screened for HIV and HCV antibodies.

The computer script, which converts frequency tables into our semi-quantitative presentation was written using the publicly available statistical software system *R* (version 2.13.0). Relevant documentation is presented in Appendix S1.

## Results

Table 1 summarizes HCV antibody prevalence among ANSPS participants by age (in quintiles) (rows) and survey years (columns) in a 5 by 16 matrix. Understanding this standard presentation requires the overall interpretation of 80 (i.e. 5×16) cells. A graphical representation of these data would be useful to identify and assess potential changes in HCV antibody seroprevalence by age across the survey years.

**Table 1:** *Percent tabulation*: percentage of people who inject drug attending needle and syringe programs since 1995 with Hepatitis C virus by age × survey years.

Age (quintiles)	1995	1996	1997	1998	1999	2000	2001	2002	2003	2004	2005	2006	2007	2008	2009	2010
<25	33%	21%	20%	22%	27%	36%	38%	44%	40%	36%	37%	40%	34%	35%	25%	25%
25–29	57%	51%	43%	38%	39%	43%	45%	47%	49%	44%	51%	54%	48%	50%	46%	43%
30–34	71%	68%	63%	57%	58%	57%	60%	57%	53%	52%	52%	51%	52%	55%	45%	49%
35–40	74%	83%	76%	71%	74%	64%	72%	69%	69%	63%	66%	62%	57%	58%	52%	54%
41 +	81%	85%	77%	68%	75%	76%	81%	72%	78%	74%	78%	77%	69%	70%	59%	62%

**Figure 1:**
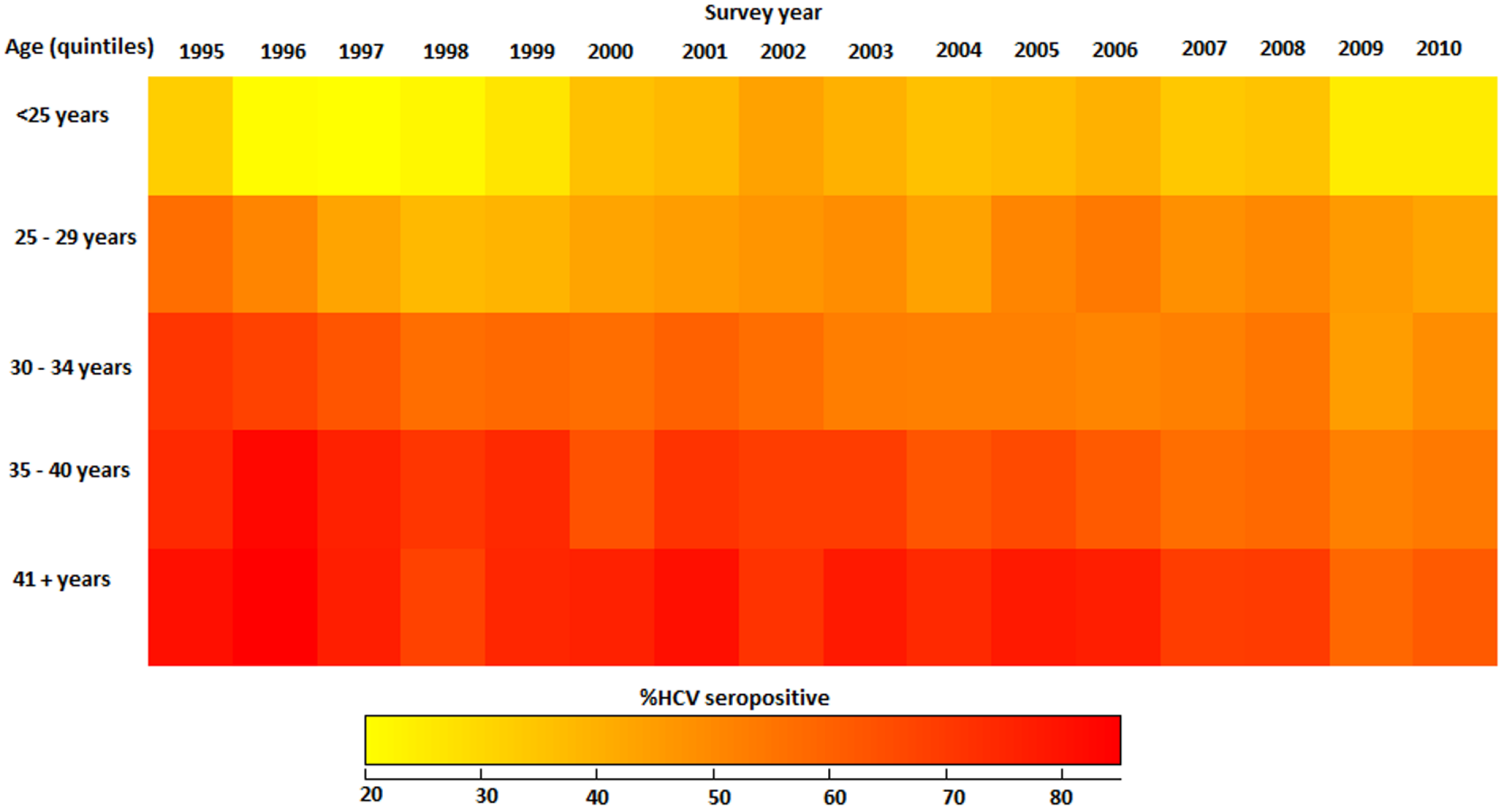
Quilt plot: percentage of people who inject drug attending needle and syringe programs since 1995 with Hepatitis C virus by age survey years.

The “*quilt plot*” (Figure 1) provides an alternative presentation of the data shown in Table 1. Here, HCV antibody prevalence by year is translated into colour levels. The colour scale changes from light to dark according to the prevalence of HCV, indicating low to high prevalence. In other words, the numbers in Table 1 have been replaced by changing colour scales that represent their magnitude, where rows correspond to age groups (quintiles) and columns correspond to survey years, and darker cells express higher HCV antibody prevalence.

A comparison of HCV antibody seroprevalence in each survey year across multiple age groups can be readily performed by reviewing the columns from top to bottom. The quilt plot reveals the year and age groups where higher HCV seropositivity was detected using shades of grey to display the different magnitudes of the percentages. Intuitively, uniform changes in the intensity (scale) of the shades from “*light*” to “*dark*” along the vertical axis will indicate uniform increases or decreases in HCV antibody prevalence across the age groups for each survey year.

Similarly, the intensity of the shades along the horizontal axis indicate the uniformity or increase/decrease in HCV antibody prevalence across survey years for each age group. For example, shades of “*light*”, followed by “*dark*”, and then “*light*”, provides an indication of non-uniform changes in the absence of a certain trend or pattern. The intensity of the shades in Figure 1 clearly shows that HCV seroprevalence uniformly increased (i.e. the colour scale changes slowly from “*light*” to “*dark*”) with the quintiles of age groups across the survey years. HCV antibody seroprevalence remained reasonably stable among participants aged less than 30 years (first two quintiles) across the survey years (i.e. no particular horizontal pattern in the colour scale).

## Discussion

In this paper we present a new plot to display large volume three dimensional data. “*Quilt plots*” and the relevant computer code provided in this paper are essentially a simple formulation of the relatively complex function “*heat maps*” [6]–[8]. Other visualization techniques, such as “Fields”, produce graphics that visually resemble “*quilt plots*” and have common elements with the methodology that we propose in our study, such as use of similar plotting, image and legend arguments. However, “Fields” cannot be used for frequency tables as it was exclusively designed for analysing spatial data using spatial statistics [5].

“Quilt plots” can be considered as a simple formulation of “heat maps”. They produce a similar graphical display to “heat maps” when the “clustering” and “dendrogram” options are turned off. In addition, “quilt plots” have several advantages over “heat maps”. Firstly, unlike “heat maps”, “quilt plots” come with easily understood R-functions (i.e. plot, legend and color). In addition, R is freely available software and supported by leading statistical experts around the world, and it is important to promote the use of this software among epidemiological researchers. In addition it is difficult to learn to use R compared to other statistical packages. For example, “heat maps” require the specification of 21 arguments including hierarchical clustering, weights for re-ordering the row and columns dendrogram, which are not always easily understood unless one has an extensive programming knowledge and skills. One of the aims of our paper is to present “quilt plots” as a useful tool with simply formulated R-functions that can be easily understood by researchers from different scientific backgrounds without high-level programming skills.

We believe this will be a valuable tool for visualisation of data particularly for multi-level categorical variables. We also provide code to produce the plots in freely available R software. The quilt plot example in this study illustrates use of percentages, however quilt plots can reveal significant features of any numerical data using the same codes, with values shown as dark to light based on absolute magnitude.

“*Quilt plots*” share some of the limitations of other graphical methods. First, they are used for data presentation rather than data analysis, and p-values are not generated. Second, they do not indicate any sense of variability measures such as standard deviation and/or confidence intervals. Graphical presentation of data, such as described in this study, may not always be an effective solution or a substitute for conventional numerical analytical tools. Nevertheless, we recommend that, where possible, the “*quilt plot*” graphical device is used along with traditional quantitative assessments of the data as an explanatory data visualization and analysis tool.

Although our method cannot be considered “new”, the novelty is to make these types of methodologies more accessible for researchers from different scientific backgrounds and without the need for strong computing skills. This will potentially increase the visibility and awareness of these types of useful statistical tools and graphical presentations.

## Conclusion

The quilt plot is very easy to comprehend and interpret. In the example presented here, HCV antibody prevalence was clearly higher among older PWID regardless of the survey year. Differences in HCV seroprevalence across the survey years by age groups can be readily identified by comparing the change in colour scale (e.g. light to dark). Quilt plots can reveal significant features of the collective data using black and white graphics, or some other binary colour system. We anticipate that this study will help stimulate the adoption of “*quilt plots*” alongside other widely used statistical packages and their subsequent widespread use in a range of fields.

## Supporting Information

Appendix S1 R-codes for “Quilt Plot”.
(DOC)
