# Correction: Quilt Plots: A Simple Tool for the Visualisation of Large Epidemiological Data

**DOI:** 10.1371/journal.pone.0093201

**Published:** 2014-03-18

**Authors:** The PLOS ONE Staff

The authors would like to provide a clarification in relation to several aspects of the article: 

While the article cites heat maps in several occasions, we appreciate that it could have been made clearer that the article is not describing a new tool for visualization of data but rather a simpler application of an existing one; we would thus like to make the following changes to the Abstract and Introduction sections: 

Abstract, first sentence of Method section should read: 

Method: We propose a simple use of an existing tool for visualization of data, known as a ''quilt plot'' (also defined as "heat maps"), that provides an alternative to presenting large volumes of data as frequency tables. 

Introduction (second paragraph, last sentence) should read: 

In the statistical literature, ''quilt plots'' (also known as ''image plots'' and "heat maps"), have been underutilised for the display of categorical data [5,6]. We would also like to clarify that the term 'quilt plot' was originally developed by Douglas Nychka -reference 5 in the article. In addition, in compliance with the journal's policy, we are providing the codes for the R functions described in the article:

http://www.plosone.org/attachments/pone.0085047.comment1.r

http://www.plosone.org/attachments/pone.0085047.comment2.r

http://www.plosone.org/attachments/pone.0085047.comment3.r

http://www.plosone.org/attachments/pone.0085047.comment5.r
