# Supplementary material for: Quilt Plots: A Simple Tool for the Visualisation of Large Epidemiological Data
Source: PLoS One. 2014 Jan 13;9(1):e85047. doi: 10.1371/journal.pone.0085047 (PMC3890282; doi:10.1371/journal.pone.0085047)

Package ‘Quilt Plots for Frequency Tables’

**Version:** 1.0-1

**Title:** Quilt Plot for Frequency Tables

**Author**s: Handan Wand <[hwand@kirby.unsw.edu.au](mailto:hwand@kirby.unsw.edu.au)>, Jenny Iversen <[jiversen@kirby.unsw.edu.au](mailto:jiversen@kirby.unsw.edu.au)>, Matthew Law <[mlaw@kirby.unsw.edu.au](mailto:mlaw@kirby.unsw.edu.au)>, Lisa Maher <[lmaher@kirby.unsw.edu.au](mailto:lmaher@kirby.unsw.edu.au) >

**Reference:** PlosOne (PONE-D-13-28088)

**Downloads**: (please see Appendix 1)

- R-function #1 (quilt)
- R-function #2 (image.legend)
- R-function #3 (blend.col)

**Description:** Given a row of
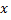
vector, a column of
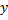
 vector and cell frequencies
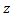
, this function converts the frequency table into a semi-quantitative presentation which plots the first variable
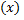
against the second variable
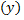
and shades the plot with respect to the third variable
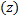
.

**Data:**

NSP.txt

The Australian Needle and Syringe Program Survey (ANSPS) [1,2]. The prevalence of hepatitis C virus (HCV) antibody among ANSPS participants were presented by age (in quintiles) (rows) survey years (columns) in a matrix.

**Format:**

This data frame contains:

- age in quintiles (row)
- Survey years (columns)
- Prevalence of HCV seropositivity (cell)

**Usage:**

percs <- t(read.csv(“NSP”)[,-1])

quilt(percs,

xlabels =dimnames(percs)[[1]],

ylabels =dimnames(percs)[[2],

zlabels =”percentage”,

col = blend.col(“white”, “black”))

**References:**

1. Iversen J, Topp L, Maher L. Australian NSP Survey National Data Report 1995 - 2010. Prevalence of HIV, HCV and injecting and sexual behaviour among NSP attendees: Kirby Institute, University of New South Wales, Sydney, 2011.
2. Kirby Institute. HIV, viral hepatitis and sexually transmissible infections in Australia, Annual Surveillance Report 2011: Kirby Institute, The University of New South Wales, Sydney, 2011.

**R-function #1: quilt**


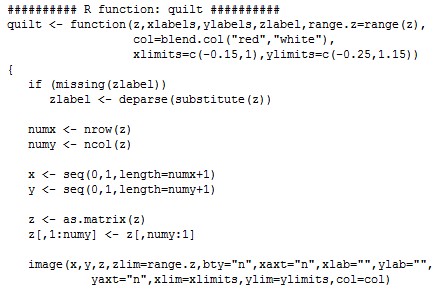


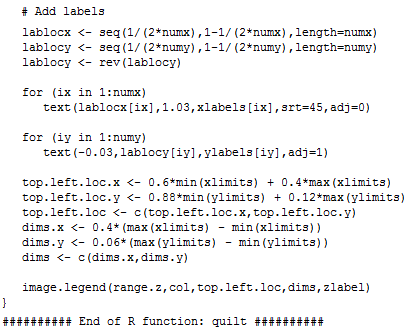


**R-function #2:** image.legend


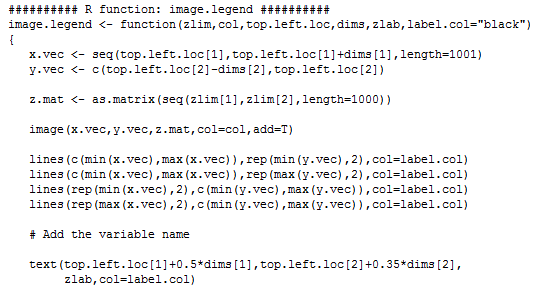


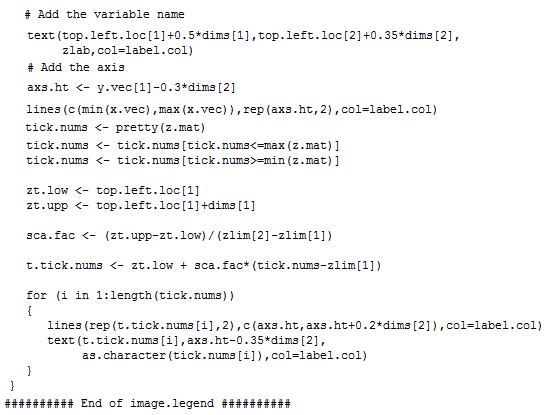


**R-function #3:** blend.col


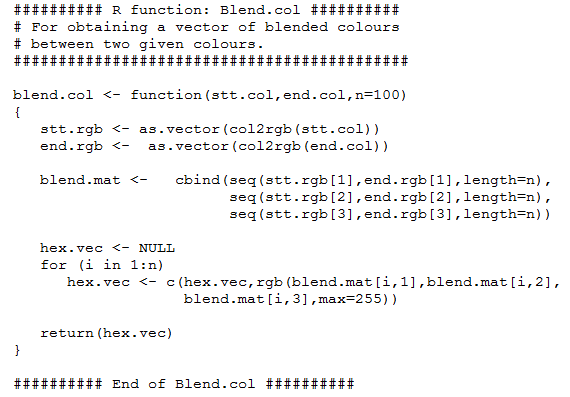

Supplement: Appendix S1 — R-codes for “Quilt Plot”. (DOC) [file pone.0085047.s001.doc]
